# Correlation of MicroRNA-31 with Endometrial Receptivity in Patients with Repeated Implantation Failure of *In Vitro* Fertilization and Embryo Transfer

**DOI:** 10.1080/15476278.2025.2460263

**Published:** 2025-02-23

**Authors:** Yan Tan, Bijun Du, Xixi Chen, Minhong Chen

**Affiliations:** a Department of Obstetrics, Guangzhou Red Cross Hospital, Guangzhou, Guangdong, China; b Department of Obstetrics, The Second Affiliated Hospital of Guangzhou Medical University, Guangzhou, Guangdong, China; c Department of Reproductive Medicine Centre, Jiangmen Central Hospital, Jiangmen, China; d Department of Obstetrics, First Dongguan Affiliated Hospital, Guangzhou Medical University, Dongguan, Guangdong, China

**Keywords:** Endometrial receptivity, endometrial thickness, *in vitro* fertilization-embryo transfer, MicroRNA-31, pulsatility index, repeated implantation failure, resistance index

## Abstract

**Objective:**

This trial probed the correlation between miR-31 expression and endometrial receptivity (ER) in patients with repeated implantation failure (RIF) of in vitro fertilization and embryo transfer (IVF-ET).

**Methods:**

A retrospective study of 80 infertility patients who underwent IVF-ET assisted conception treatment were divided into RIF group and normal pregnancy group (control group) according to the pregnancy outcome after embryo transfer. General information of both groups was collected. Endometrial tissues were collected in the middle luteal phase of the menstrual cycle before IVF-ET. miR-31 levels in endometrial tissues were measured, and endometrial tolerance indicator pulsatility index (PI), resistance index (RI), and endometrial thickness (Em) were detected. The correlation between endometrial miR-31 levels and ER indices was evaluated by Pearson method. ROC curves were utilized to analyze the efficacy of miR-31 in predicting RIF occurrence. The influencing factors of RIF were analyzed by binary Logistic regression.

**Results:**

RIF patients had increased miR-31 expression level and endometrial tolerance indicator PI, and RI while decreased Em (*p* < 0.05). miR-31 in RIF patients was positively correlated with PI and RI, and negatively correlated with Em (*p* < 0.05). The area under the curve for miR-31 to predict the occurrence of RIF was 0.899, with a sensitivity of 0.750 and a specificity of 0.950. PI, RI, and miR-31 were risk factors for developing RIF in IVF-ET women, and Em was a protective factor (*p* < 0.05).

**Conclusion:**

miR-31 in RIF patients is positively correlated with PI and RI, and negatively correlated with Em.

## Introduction

*In vitro* fertilization-embryo transfer (IVF-ET) is becoming increasingly significant in the field of infertility treatment.^1^ During pregnancy, an embryo capable of implanting and an endometrium that facilitates implantation must communicate synchronistically and coordinatedly.^2^ The human endometrium is a dynamic tissue that undergoes periodic remodeling during the menstrual cycle, becoming a recipient of embryo implantation for only a short time during each cycle.^3^ Endometrial receptivity (ER) deficiency, defined as the ability of the endometrium to receive and accommodate a newborn embryo, causes approximately two-thirds of implantation failure.^4^ Repeated implantation failure (RIF) after IVF is a challenging issue for clinicians and may be a devastating reality for some infertile patients.^5^ At present, accurate prediction tools for ER are urgently needed to accurately guide the success of embryo implantation.^6^

Ultrasound technology is receiving increasing attention due to its advantages of real-time monitoring, non-invasiveness, and predictability. The endometrial thickness, pattern, and endometrial and subendometrial blood flow measured by ultrasound scanning may be associated with ER.^7^ A class of biomarkers with potential clinical applications is microRNAs (miRNAs), which are involved in the regulation of the window of implantation timing (a narrow time frame of maximal ER).^8^ miRNAs are capable of inhibiting or promoting translation of mRNAs at the post-transcriptional level.^9^ miRNAs provide a new mechanism for embryo-maternal communication and can be used as noninvasive biomarkers for ER assessment in assisted reproduction, improving the accuracy of assessment while reducing mechanical damage to tissues.^10^ Several miRNAs such as miR-455-3p and miR-152-3p have been identified to be associated with ER status and implantation failure and are of value in predicting pregnancy outcome.^11^ miR-182-5p is upregulated during the secretory phase in infertile women and therefore can be a potential biomarker for negative selection in ER.^12^ Moreover, miRNAs have been reported in the endometrium, and these miRNAs may potentially regulate ER.^13,14^ miR-31 is a potential biomarker of optimal ER and may act through immunosuppressive mechanisms.^15^ miR-31-3p expression has been demonstrated to be elevated in the endometrium of patients with cesarean scar endometriosis.^16^ However, the correlation between miR-31 and ER waits for comprehensive analysis in patients with RIF of IVF-ET.

This trial, therefore, conducted a correlation study on the relationship between miR-31 in the endometrium with ER-related indices in patients experiencing IVF-ET. It is hoped to validate the possibility of miR-31 as an indicator to predict RIF.

## Materials and methods

### Ethical approval

This retrospective study was approved by the Ethics Committee of Guangzhou Red Cross Hospital (approval number: 2017093) and informed consent was waived.

### Participants

This study was a retrospective analysis of the clinical data of infertility patients who underwent IVF-ET assisted conception treatment in Guangzhou Red Cross Hospital from January 2018 to December 2020. The patients were divided into a RIF cycle group (RIF group) and a normal pregnancy cycle group (control group) according to the pregnancy outcome. Inclusion criteria for the RIF group: the female partner was <40 years old and failed to achieve a clinical pregnancy after the transfer of at least 3 good-quality embryos in 3 cycles of fresh or frozen-thawed embryo transfer, of which the good-quality embryos included good-quality cleavage-stage embryos on the third day (≥8 cells, uniform size of blastomere, and fragmentation rate of <10%) and high-quality blastocysts (≥3BB). Inclusion criteria for the control group: female partner’s age <40 years old, clinical pregnancy obtained in one embryo transfer cycle.

Exclusion criteria: 1. Women with chromosomal abnormalities; 2. Women had a clear history of mental illness; 3. Women with genital malformation, endometritis, endometrial polyp, or polycystic ovary syndrome; 4. Women with abnormal pituitary or thyroid function affecting sex hormone levels; 5. Women with serious dysfunction of heart, lung, kidney, and other organs; 6. Women complicated with immune deficiency disease, acute and chronic hepatitis, and malignant tumor.

A total of 40 RIF cycles and 40 normal pregnancy cycles were included.

### General clinical data

Age, years of infertility, body mass index (BMI), number of miscarriages, HCG daily estradiol (E2) and progesterone (P) levels were collected from the patients.

### Collection and preservation of endometrial tissue samples

Endometrial tissues were collected 1 week after ovulation, i.e., mid-luteal phase of the menstrual cycle 1 week prior to IVF-ET using disposable uterine tissue suction tubes. The endometrium was obtained by scratching near the uterine fundus. After removal of the endometrial tissue, it was quickly and gently rinsed with saline to avoid contamination with blood, which was then stored at −80°C for later use. Endometrial tissue specimens were completed by the same physician who had experience in this field.

### Detection of miR-31 in endometrial tissues

Total RNA was extracted from endometrial tissues by Trizol method (Invitrogen, Carlsbad, CA, USA). The purity and concentration of total RNA were determined by NanoDrop-2000 spectrophotometer. About 1 μg of RNA was reverse transcribed to cDNA with a miRNA reverse transcription kit (from miScript II RT kit Qiagen, Hilden, Germany). miR-31 expression level was evaluated using SYBR green (miScript SYBR Green PCR kit, Qiagen, Hilden, Germany) for real-time PCR following the manufacturer’s protocols. miR-31 primer (5′-AGGCAAGATGCTGGCATAGCT-3′); U6 (forward: 5′-GGAACGATACAGAGAAGATTAGC-3;′ reverse: 5′-TGGAACGCTTCACGAATTTGCG-3′). The reaction conditions were: 95°C for 10 min, and 40 PCR cycles were performed (95°C for 10 s; 60°C for 30 s). U6 served as an endogenous control for miR-31. The 2^−ΔΔCt^ method was adopted to normalize and calculate fold changes in miR-31 expression. Delta Ct (ΔCt) values for miR-31 were attained after normalization to U6, ΔΔCt = ΔCt (RIF group) - ΔCt (control group).

### ER test

On the day of embryo transfer, ER was detected by vaginal B-ultrasonography. A SIEMENS ACUSONx700 ultrasound diagnostic machine was used, and the intracavitary probe was an EC9-4W. Depth and gain were adjusted to make the endometrial layers clear when observing with two-dimensional ultrasound, and when measuring the parameters of the spiral arteries of the uterus with spectral Doppler (PW), the angle of the calibrated blood flow to the acoustic beam was ≤60°, and the sample line was narrowed down to a suitable size of 1–2 mm. Measurements were standardized by two experienced physicians. Endometrial thickness, uterine spiral artery pulsatility index (PI) and resistance index (RI) were measured by transcavitary ultrasound. 1. Endometrial thickness: The rotating probe is set so that the image is presented in the sagittal plane of the center of the endometrium, and the thickness of the endometrium is measured at a distance of 2 cm from the uterine fundus (with the average of two measurements). 2. Uterine spiral artery RI (peak systolic blood flow rate-end diastolic blood flow rate/peak systolic blood flow rate), PI (peak systolic blood flow rate-end diastolic blood flow rate/average rate) measurement: The color Doppler mode is selected, the sampling frame will be completely wrapped around the endometrium and part of the adjacent myometrial layer, the clearer vessel is selected for the spectral Doppler (PW) to measure the relevant parameters (three or more consecutive clear spectra are required for measurement).

### Statistical analysis

SPSS26.0 statistical software was used for data analysis. Enumeration data were expressed as cases (%) and χ^2^ test was applied for comparative analysis. Measurement data in normal distribution were expressed as $\left(\overset{ˉ}{X}\pm S\right)$, and t-test was applied for comparison between the two groups. Measurement data that did not fit the normal distribution were expressed as M (P25, P75) and compared with the Mann-Whitney U test. The receiver operating characteristic (ROC) curve was plotted to evaluate the efficacy of miR-31 in predicting RIF. The correlation between ER and miR-31 levels in RIF patients was analyzed by the Pearson method. The influencing factors of RIF were analyzed by binary Logistic regression analysis. *p* < 0.05 indicated that the difference was statistically significant.

## Results

### General data

Age, BMI, infertility years, abortion history, HCG daily E2 and P levels were not significantly different between the RIF and control groups (*p* > 0.05) (Table 1).

**Table 1.** Comparison of general information between the two groups.

Indicators	Control group	RIF group	*P* value
	(*n* = 40)	(*n* = 40)	
Age $\bar{X}\pm S$, years)	32.60 ± 3.56	33.93 ± 3.02	0.077
Body mass index $\left(\overset{ˉ}{X}\pm S\right)$, kg/m^2^	24.65 ± 3.51	25.21 ± 3.62	0.484
Infertility years (x±s, years)	4.37 ± 2.00	5.04 ± 1.82	0.145
History of abortion [n (%)]			
Yes	24 (60.00)	28 (70.00)	0.348
No	16 (40.00)	12 (30.00)	
HCG daily estradiol [M (P25, P75), ng/ml]	2213 (1903, 3296)	2752 (1795, 5879)	0.063
HCG daily progesterone $\left(\overset{ˉ}{X}\pm S\right),\mathrm{nmol}/L)$	0.82 ± 0.31	0.93 ± 0.32	0.123

RIF, repeated implantation failure. Measurement data that conformed to normal distribution were analyzed by t-test and those that did not fit the normal distribution were analyzed by Mann-Whitney U test. Enumeration data were compared by χ^2^ test.

### miR-31 levels in endometrial tissues

Table 2 shows higher endometrial miR-31 expression in the RIF group than in the control group (*p* < 0.05).

**Table 2.** Comparison of miR-31 levels in endometrial tissues between the two groups.

Groups	miR-31
Control group (*n* = 40)	1.02 ± 0.30
RIF group (*n* = 40)	1.97 ± 0.70
*P* value	<0.001

RIF, repeated implantation failure. Measurement data that conformed to normal distribution were analyzed by t-test.

### ER indices

Endometrial PI and RI in the RIF group were higher and Em was lower than those in the control group (*p* < 0.05, Table 3).

**Table 3.** Comparison of endometrial receptivity indices between the two groups $\left(\overset{ˉ}{X}\pm S\right)$.

Groups	PI	RI	Em (mm)
Control group (*n* = 40)	1.33 ± 0.33	0.71 ± 0.20	10.68 ± 2.56
RIF group (*n* = 40)	1.98 ± 0.58	0.89 ± 0.26	8.64 ± 1.57
*P* value	<0.001	<0.001	<0.001

RIF, repeated implantation failure; PI, pulsatility index; RI, resistance index; Em, endometrial thickness. Measurement data that conformed to normal distribution were analyzed by t-test.

### Evaluation of the predictive value of miR-31 and endometrial tolerance indices (PI, RI, and Em) for RIF

**Figure 1.**
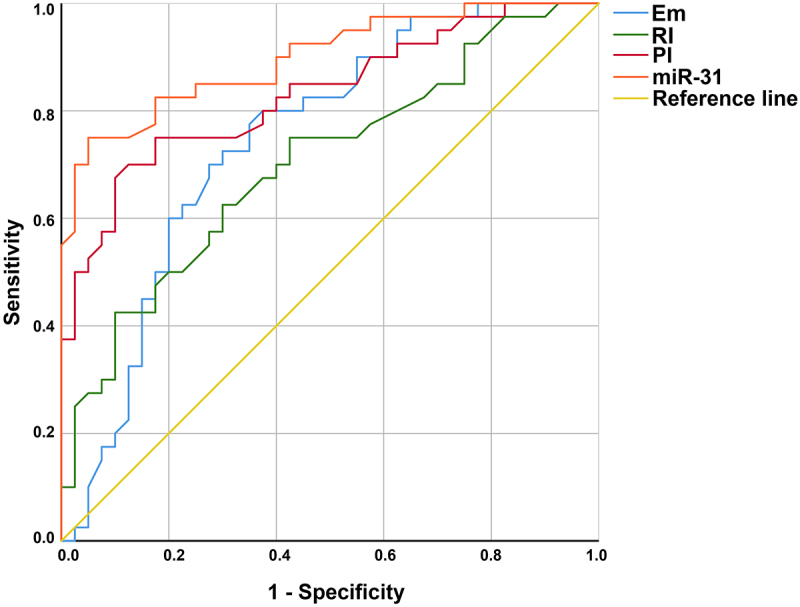
ROC curve analysis of endometrial tissue miR-31 and endometrial tolerance indices (PI, RI, and Em) for prediction of RIF.

The diagnostic value of miR-31 for RIF (RIF group and control group) was analyzed using ROC curves. ROC curve analysis showed that the AUC for miR-31 to predict RIF was 0.899, with an optimal cutoff value of 1.52, a sensitivity of 75% and a specificity of 95%. The AUC of PI, RI, and Em for predicting RIF were 0.832, 0.703, and 0.747, respectively, and the optimal cutoff values were 1.62, 0.73, and 9.39, respectively, with sensitivities of 75.0%, 75.0%, and 70.0% and specificities of 82.5%, 57.5%, and 72.5%. (Table 4 and Figure 1).

**Table 4.** Assessment of the value of miR-31 and endometrial tolerance indicators (PI, RI, and Em) in predicting RIF.

Indicator	AUC	Cut-off	Sensitivity (100%)	Specificity (100%)	Youden index	*P* value	95%CI
miR-31	0.899	1.52	75.0%	95.0%	0.700	<0.001	0.831 ~ 0.966
Em	0.747	9.39 (mm)	70.0%	72.5%	0.425	<0.001	0.637 ~ 0.856
PI	0.832	1.62	75.0%	82.5%	0.575	<0.001	0.743 ~ 0.921
RI	0.703	0.73	75.0%	57.5%	0.325	0.002	0.589 ~ 0.817

RIF, repeated implantation failure; IVF-ET, *in vitro* fertilization and embryo transfer. ROC curve was plotted to evaluate the diagnostic efficacy.

### Correlation analysis of endometrial miR-31 levels and ER in patients with RIF

The correlation between miR-31 expression in endometrial tissue and endometrial tolerance indices (PI, RI, and Em) in patients with RIF was analyzed. Pearson correlation analysis (Table 5) revealed that endometrial miR-31 in RIF patients was positively correlated with PI and RI (*r* = 0.434, *p* = 0.005; *r* = 0.515, *p* < 0.001) and was negatively correlated with Em (*r* = −0.603, *p* < 0.001).

**Table 5.** Correlation analysis between endometrial miR-31 levels and endometrial receptivity indices in patients with RIF.

	miR-31	
	r	*P* value
PI	0.434	0.005
RI	0.515	<0.001
Em	−0.603	<0.001

RIF, repeated implantation failure; PI, pulsatility index; RI, resistance index; Em, endometrial thickness. Pearson test was used for correlation analysis.

### Influencing factors of RIF

With the occurrence of RIF as the dependent variable and miR-31, PI, RI, and Em as independent variables, a binary Logistic regression analysis was performed (Table 6). PI, RI, and miR-31 were risk factors and Em was a protective factor affecting RIF in IVF-ET women (all *p* < 0.05).

**Table 6.** Influencing factors of RIF were analyzed by binary Logistic regression analysis.

Indices	β	SE	Wales	*P*	OR	95%CI
PI	3.212	0.751	18.296	<0.001	24.831	5.699 ~ 108.197
RI	3.466	1.125	9.496	0.002	31.995	3.53 ~ 289.975
Em	−0.469	0.133	12.383	<0.001	0.626	0.482 ~ 0.812
miR-31	4.103	0.929	19.519	<0.001	60.541	9.806 ~ 373.783

RIF, repeated implantation failure; PI, pulsatility index; RI, resistance index; Em, endometrial thickness. Logistic regression analysis was used.

## Discussion

Implantation is a critical step in human reproduction that relies on a capable blastocyst, an acceptable endometrium, and a successful crosstalk between the embryo and the maternal interface. RIF, which refers to implantation failure after multiple embryo transfers, has become unacceptable for patients and providers.^17^ Both high-quality embryos and synchronous endometrium are important. How to obtain the optimal ER is a challenge for implantation and pregnancy in infertile patients with RIF.^18^ Therefore, identifying risk factors for RIF and improving ER in patients with IVF-ET have become challenging tasks in clinical settings. Based on this, this trial probed the values of miR-31 and its association with ER in RIF patients and eventually identified that miR-31 expression in the endometrium of RIF patients was increased, which was positively correlated with PI and RI, and negatively correlated with Em.

miR-31 has been classified as a potential biomarker for optimum receptivity that is significantly elevated in the secretory phase in human endometrium during the window of implantation.^15^ Meanwhile, has-miR-31 upregulation was once detected in the proliferative phase of RIF patients, suggesting its diagnostic potential for RIF.^19^ This trial also measured an upward trend in miR-31 expression in the endometrium of RIF patients. Moreover, according to ROC analysis curves, miR-31 could predict the occurrence of RIF with high specificity and sensitivity. It is suggested that miR-31 has predictive values in patients receiving IVF-ET to suffer from RIF. Previous studies have generally discussed miR-31’s action in the ovary. For instance, miR-31 targets FSH reporter gene to regulate steroid hormone metabolism/synthesis and apoptosis of ovarian granulosa cells.^20,21^ Meanwhile, miR-31 has the potential to act as biomarkers for guiding diagnosis and assessing prognosis and metastatic process in breast cancer patients.^22^ Moreover, miR-31-3p acts as a potent therapeutic target and new prognostic indicator for cervical cancer patients, and miR-31-3p could be serve as a new method to reverse the chemotherapy resistance in cervical cancer.^23^ The functional role of miR-31 is complicated and miR-31 can show tumor suppressive and oncogenic roles in diverse tumor types. The phenotype resulting from aberrant miR-31 expression is strongly dependent on its endogenous expression levels.^24^ However, much remains to be delved into its action in assisted production and complicated reproduction failure.

The uterine spiral artery is the terminal branch of the uterine artery and the main blood vessel that feeds the endometrium and provides nutrients for fetal growth.^25^ Successful implantation of the embryo requires a receptive endometrium. Accordingly, Em is the most commonly used indicator of ER during assisted reproduction,^26^ while RI and PI assessments are applicable to evaluate ER in an assisted reproduction program.^27^ In clinical cases, poor ER is often accompanied by elevated RI and PI values of endometrial blood flow and reduced Em.^28,29^ This trial evaluated ER in RIF patients by measuring RI, PI, and Em and determined that PI and RI of the uterine spiral artery in RIF patients were higher and Em was lower in RIF patients compared to controls, indicating poor ER in the patients.

Correlation analysis found that endometrial miR-31 in RIF patients was positively correlated with PI and RI and was negatively correlated with Em, and all these indices were independent risk factors of RIF in patients after IVF-ET. It is notably addressed that higher PI and RI for endometrial blood flow are available indices to predict unexplained RIF,^30^ and RI is independently associated with ET failure and it is used as a component of a diagram that has been developed to visualize the likelihood of IVF-ET implantation failure.^31^ However, no published reports have pointed out the correlation between miR-31 and these ER-associated indices.

In summary, endometrium miR-31 expression was increased in RIF patients, and this was positively associated with PI and RI, and negatively associated with Em. Given that, the detection of miR-31, PI, RI, and Em is of predictive value for RIF in patients with IVF-ET. This trial only enrolled 40 cases, and a relatively small size may produce results bias. Therefore, the application values of these indices need to be checked and validated in future analyses.

## Data Availability

The original contributions presented in the study are included in the article/Supplementary Material, further inquiries can be directed to the corresponding author.
